# The Effect of Advanced Maternal Age on Embryo Morphokinetics

**DOI:** 10.3389/fendo.2019.00686

**Published:** 2019-10-25

**Authors:** Miriam Warshaviak, Yael Kalma, Ariela Carmon, Nivin Samara, Michal Dviri, Foad Azem, Dalit Ben-Yosef

**Affiliations:** ^1^IVF Lab and Wolfe PGD-Stem Cell Lab, Fertility Institute, Tel-Aviv Sourasky Medical Center, Tel Aviv, Israel; ^2^Department of Cell Biology and Development, Sackler Faculty of Medicine, Tel-Aviv University, Tel Aviv, Israel

**Keywords:** embryo development, advanced maternal age (AMA), time lapse microscopy, morphokinetics, *in vitro* fertilization (IVF)

## Abstract

**Purpose:** To compare the morphokinetic parameters of pre-implantation development between embryos of women of advanced maternal age (AMA) and young women.

**Methods:** Time-lapse microscopy was used to compare morphokinetic variables between 495 embryos of AMA women ≥ age 42 years and 653 embryos of young patients (<age 38 years) who underwent IVF in our unit. Developmental events annotated and analyzed include observed cell divisions in correlation to the timing of fertilization, synchrony of the second (s2) and third cell cycles (s3) and the duration to the second (cc2) and third cleavages (cc3).

**Results:** No significant differences were observed in cleavage times between the embryos of AMA and the control embryos. Interestingly, the older embryos appear to be more prone to developmental arrest (a higher percentage of embryos of older women arrested at 4–7 cells resulting in less embryos reaching the 8-cell stage (66% vs. 72%, respectively), though this difference did not reach a significance at least during the first 3 days of development (*p* > 0.05).

**Conclusions:** While early morphokinetic parameters do not reflect dynamics unique to embryos of older women, a tendency toward developmental arrest was observed, which would likely be even more pronounced at later stages of development.

## Introduction

Reproductive capacity in women declines dramatically with advancing age (1). Spontaneous cumulative pregnancy rates begin to decline at age 35–39 and approach almost zero soon after 45 years (2, 3). In accordance, a decrease in pregnancy and delivery rates is correlated with increased age in women undergoing *in vitro* fertilization (IVF) treatment (2–7).

Increasing age is associated with a decrease in both oocyte quantity and quality (8, 9), and an increased rate of chromosomal aneuploidy (1, 8, 10), which correlates with lower fertilization rates, poorer embryo development, and decreased implantation and pregnancy potential (10). Studies on donor oocytes of younger women revealed similar implantation, miscarriage and delivery rates among donor oocyte recipients of different ages, suggesting that decreased fertility in advanced age is attributable mainly to oocyte quality rather than to uterine/endometrial aging (11, 12).

Pre-implantation genetic screening (PGS) that provides information on embryo ploidy can affect IVF outcomes (10), but the use of this invasive and costly procedure remains a matter of controversy, as its value in increasing take-home baby rates or cumulative delivery rates per patient remains to be proven (13–15).

In the clinical IVF laboratory setting, embryo evaluation by morphologic criteria at distinct time points (static evaluation) is still considered the most useful non-invasive tool for selecting embryos with the highest implantation potential (16–18). However, it is limited in its ability to capture the continuous dynamic process of embryonic development. In addition, numerous large studies concluded that the routinely used static morphological scoring systems fail to reflect age-related impact on oocyte and embryo quality (19–21).

In the search for new parameters, time-lapse microscopy (TLM) presents an opportunity for optimizing embryo selection based on kinetic evaluation which may further improve selection of viable embryos. Using various endpoints, including blastocyst formation, implantation success, and take-home baby rates, logistic regression models have been used to identify morphokinetic parameters which have been considered as being favorable to implantation (22–25). In addition, deselecting embryos with uneven blastomere size, direct division from one to three cells, and <8 cells at 68 h post-insemination have also been identified as being prognostic for embryo implantation (24, 26), and demonstrated improved outcomes compared to those using static evaluation alone (27–29).

However, limited data is available regarding the impact of maternal age on embryo morphokinetics. Gryshchenko et al. recently found that the woman's age had no effect on the kinetic parameters of embryo development, but this was based on an analysis of only 86 embryos of women >40 years of age (30). Another study focused primarily on the correlation between morphokinetics and ovarian reserve demonstrated that the time from insemination to first cleavages in embryos produced by a subgroup of women with normal ovarian reserve was significantly longer in those of “older” women (30–40 years) compared to those of younger women (20–30 years). However, in this study no data were provided on embryos of women >40 years of age (31).

We here conducted the first large-scale systematic analysis that aims to compare the morphokinetic parameters of early embryonic development of embryos from women of advanced maternal age (AMA, ≥42 years) to those of younger women (YW, <38 years of age).

## Materials and Methods

### Study Population and Design

This study is a retrospective data analysis. The study group consisted of all embryos from women ≥42 years of age (the AMA group) that underwent IVF treatment consisting of standard insemination (51 women, 83 cycles, 495 embryos) at our unit from September 2012 to December 2014. Inclusion criteria included all embryos produced by standard insemination (IVF), incubated in the EmbryoScope™ and either transferred, cryopreserved or discarded. ICSI embryos were excluded to focus solely on factors attributed to female age that could affect embryonic development and to rule out male factor. Only treatments in which all embryos (including those transferred, frozen or discarded) were incubated in the EmbryoScope™ for a minimum of 72 h were included in the study. Treatments in which embryos were transferred or frozen at an earlier stage were excluded so that they would not be confused with those who were included in the study and whose development had arrested. Excluded were all embryos created with intracytoplasmic sperm injection (ICSI), when the indication for treatment was of PGD or fertility preservation, embryos produced during the study period but not incubated in the EmbryoScope ™, embryos for which annotation was impossible (e.g., degenerative embryos), and embryos from cycles in which no embryos were transferred (Supplemental Figure 1).

The control group included all embryos of women <38 years of age (the YW group) from cycles performed during the same time period, that underwent standard insemination (not ICSI), and were cultured in the EmbryoScope™ at least until day 3 (71 women, 82 cycles, 653 embryos) (Supplemental Figure 2).

### Ovarian Stimulation, Fertilization, and Embryo Culture

Controlled ovarian stimulation was carried out by the long gonadotropin-releasing hormone (GnRH) agonist, short GnRH agonist, or GnRH antagonist protocols. The long protocol began with the administration of subcutaneous injections of 0.1 mg/d of the GnRH-α triptorelin (Decapeptyl; Ferring, Kiel, Germany) for at least 14 days, followed by concomitant recombinant follicle-stimulating hormone [rFSH; Gonal F (Serono, Geneva, Switzerland) or Puregon (Organon, Oss, The Netherlands)] or human menopausal gonadotrophin (hMG; Menogon, Ferring, Kiel, Germany) or highly purified human menopausal gonadotropin (Menopur, Ferring Pharmaceuticals, Geneva, Switzerland). The short protocol began with the administration of the GnRH-α from the first day of the cycle followed by concomitant daily r-FSH and GnRH-α from day 3 of the cycle. In the antagonist protocol, the stimulation protocol started with administration of gonadotropins from day 2 to 3 of the cycle. GnRH antagonist (0.25 mg of cetrorelix acetate, Cetrotide®, Serono or ganirelix, Orgalutran®, Merck and Co, Inc.) administration was started when the leading follicle exceeded ≥12 mm or the estradiol level was >450 pg/ml and continued until the day of human chorionic gonadotropin (hCG) administration. Choriogonadotropin alfa 250 mcg (Ovitrelle; Serono, Geneva, Switzerland) was administered when at least three follicles achieved an 18-mm diameter. Ovum pickup was performed 36 h later.

The cumulus-oocyte complexes were isolated into multipurpose handling medium-complete (MHM-C) (Irvine Scientific). Sperm samples were treated with MHM-C medium (Irvine Scientific). Insemination was performed 2–4 h following oocyte retrieval. Embryos were denuded of cumulus cells by fine pipette 20–22 h post-insemination. Each embryo was incubated in a separate droplet of human embryo culture medium (SAGE 1-Srep, Origio or GT RONI) covered with paraffin oil (Oil for Embryo Culture, Irvine Scientific) in the EmbryoSlide® culture dish (Fertilitech) to allow individual assessment and documentation. Incubation in the EmbryoScope™ incubator (5% O_2_, 5.5% CO_2_, temperature level 37.0°C) lasted from day 1 following IVF and continued up to day 3 of development.

### Time-Lapse Monitoring of Embryo Morphokinetics

All embryos were incubated in the integrated EmbryoScope™ time-lapse monitoring system (EmbryoScope™; UnisenseFertiliTech, Vitrolife Denmark,) from the time of fertilization until day 3 of embryonic development. The EmbryoScope™ offers the possibility of continuous monitoring of embryo development without disturbing the culture conditions. Embryo scoring and selection with time-lapse monitoring were performed by analysis of time-lapse images of each embryo on an external computer by means of software developed specifically for image analysis (EmbryoViewer workstation; Unisense Fertilitech A/S Vitrolife). Embryo morphology and developmental events were recorded to demonstrate the precise timing of the observed cell divisions in correlation to the timing of fertilization: time of pronuclei fading (tPnf); cleavage to a 2-blastomere (t2), 3-blastomere (t3), 4-blastomere (t4), and so forth until reaching an 8-blastomere (t8) embryo. In addition, the synchrony of the second (s2) and third cell cycles (s3) and the duration to the second (cc2) and third cleavages (cc3) were measured. All cleavage times (t2–t8) were standardized with respect to the tPNf. All the assessments and annotations of the embryos were performed by senior embryologists, ensuring a very low inter-observer variation.

### Statistical Analysis

The normal distribution of the embryo morphokinetic parameters was checked by the Kolmogorov-Smirnov test. As all measures demonstrated abnormal distribution, they are reflected by median and interquartile ranges (Q1, Q3). In order to compare the parameters of the different age groups, each parameter was ranked and the rank transformed values were used for the statistical test.

The comparison of embryo parameters between the age groups was done by the generalized linear model Glimmix with the Gaussian link function for the morphokinetic parameters and with the Logit link for comparing whether or not the parameter exists for individual embryos. The Glimmix regression model performs a double adjustment of the embryonic data that takes into account the biological dependency of embryos to both the treatment cycle and the woman, accounting for the two-level hierarchical structure of the data: embryos clustered within treatment, treatment clustered within women.

Comparing treatment ART outcomes between age groups was done by the generalized linear regression model Glimmix as well as with the Logit link. This comparison was adjusted to account for the biological dependency of treatments from the same women.

The chi-square test was used to compare the indications for IVF between the two groups.

A *P* < 0.05 was considered significant. Statistical analysis was performed by SAS for windows version 9.4.

### Ethical Approval

This study is a retrospective data analysis. The clinical database was approved for research purposes by the National Justice Ministry (#980044213). In addition, this study was approved by the Ethics Committee of Tel Aviv Medical Center, and institutional review board for retrieving IVF data (0606/17).

## Results

A total of 1,148 IVF embryos were evaluated, including 495 from women in the AMA group and 653 in the YW group. Of these 1,148 embryos, 430 (37%) were transferred following fresh treatment cycles, 196 (17%) were frozen and 522 (46%) were discarded due to very low quality.

The embryos were derived from 122 women who underwent 165 treatment cycles, including 51 AMA subjects (83 treatments) and 71 YW subjects (82 treatments) (Table 1). Most of the women in the AMA group had unexplained infertility, probably related to their age (i.e., the sperm parameters were normal), while most of the women in the YW group had either unexplained (most likely female factor) or mechanical infertility, both of which are the most common indications for IVF using standard insemination (Table 1).

**Table 1:** Indications for *in vitro* fertilization in the advanced maternal age (study) and younger women (control) groups.

**Indication for IVF**	**AMA (Age ≥ 42)**	**YW (Age < 38)**	***p*-value**
Number of women	51	71	
Unexplained infertility	82% (42)	54% (38)	*p* < 0.001
Mechanical factor	14% (7)	39% (28)	*p* < 0.05
Anovulation	2% (1)	1% (1)	NS
Endometriosis	2% (1)	6% (4)	NS

*AMA, advanced maternal age ≥ 42 years (study group); YW, young women < age 38 years (control group). The Chi-square test is used to compare proportions between the groups*.

There was no group difference in the number of previous failed IVF cycles (Table 2). As expected, significantly fewer oocytes were retrieved among the AMA women compared to the controls (8 vs. 12.5, respectively; *p* < 0.001). The fertilization rate was similar in both groups, but, as expected, ongoing pregnancy rates defined by the presence of a fetal heartbeat (11% vs. 28% for the YW group, *P* < 0.01) and live birth rates (2% vs. 27% in the YW group; *P* < 0.01) were significantly lower in the AMA group compared to the young patients (Table 2).

**Table 2:** Cycle characteristics and ART outcomes.

	**AMA (Age ≥ 42)**	**YW (Age < 38)**	***P* value (adjusted)**
Number of cycles	83	82	
Fertilization rate	80%	70%	NS
Median (Q1, Q4)	(60%, 90%)	(60%, 90%)	
Number of embryos	495	653	
*Transferred (%)*	52%	27%	
*Frozen (%)*	6%	25%	
*Discarded (%)*	42%	48%	
Previous failed IVF cycles (Median (Q1, Q4)	2 (1.0, 5.0)	2 (0.0, 4.0)	NS
Oocytes aspirated (Median (Q1, Q4)	8.0 (7.0, 10.0)	12.5 (7.5, 15.0)	<0.0001
Positive bhCG/ET (%)	18%	35%	0.017
Ongoing pregnancy rate (fetal heartbeat) (%)	11%	28%	0.01
Live birth rate (%)	2.4%	27%	0.0009

*AMA, advanced maternal age ≥ 42 years (study group); YW, young women < age 38 years (control group). The Wilcoxon sum ranked test was used for comparing between the study and control groups and the Chi square test to compare proportions between the groups. Data is presented as median and interquartile range (Q1, Q3)*.

**Figure 1:**
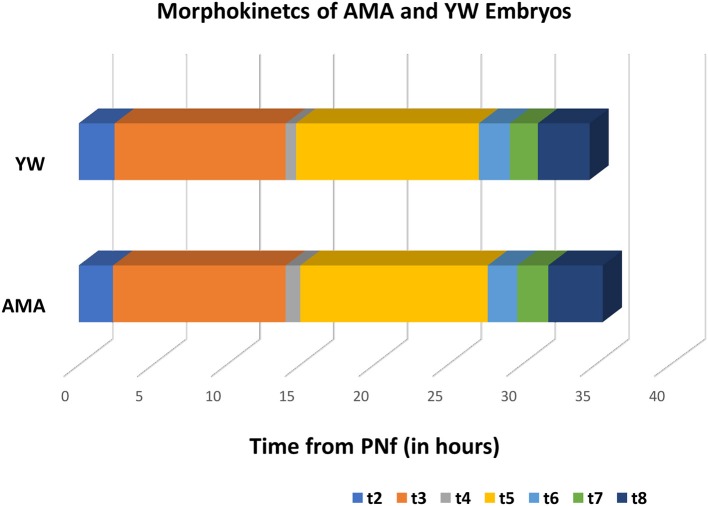
A comparison of the morphokinetic parameters of AMA and YW embryos.

In order to compare the morphokinetic parameters of embryos of AMA patients with that of young patients, all embryos with visible and annotated PN fading were analyzed (1,087 embryos which constitute 94% of the total of 1,148 embryos included in the study). No significant differences were observed in all early pre-implantation morphokinetic parameters analyzed until embryo transfer at day 3 (cleavage times, synchronicity of the cell cycles, and time to second and third cleavage), between the study and control groups (Table 3 and Figure 1).

**Table 3:** Comparison between the morphokinetic parameters of embryos of aged and young women.

Parameter^*^ (relative to tPNF^**^)	**Age** **≥** **42 (*****N*** **=** **467)**		**Age** **<** **38 (*****N*** **=** **611)**		***P* value**
	**Median**	**Q1, Q40**	**Median**	**Q1, Q40**	
t2	2.3	(2.0, 3.0)	2.4	(2.1, 3.0)	0.45
t3	14	(13.0, 15.7)	14	(12.7, 15.0)	0.41
t4	15	(13.7, 16.7)	14.7	(13.7, 16.6)	0.67
t5	27.7	(24.1, 32.1)	27.1	(23.4, 30.0)	0.09
t6	29.7	(26.3, 33.8)	29.2	(26.3, 33.3)	0.66
t7	31.8	(28.2, 37.9)	31.1	(28.2, 37.3)	0.78
t8	35.5	(30.4, 42.0)	34.6	(30.0, 42.3)	0.87
cc2 (t3-t2)	11.5	(10.3, 13.0)	11.4	(10.3, 12.3)	0.42
s2 (t4-t3)	0.7	(0.3, 1.7)	0.7	(0.0, 1.3)	0.92
cc3 (t5-t3)	14	(11.4, 17.0)	13.5	(11.4, 15.7)	0.1
s3 (t8-t5)	7	(3.7, 15.4)	8	(3.7, 16.0)	0.4

* *AMA, advanced maternal age ≥ 42 years (study group); YW, young women < age 38 years (control group); t2, time to cleavage into 2 cells; t3, time to cleavage into 3 cells; t4, time to cleavage into 4 cells; t5, time to cleavage into 5 cells; t6, time to cleavage into 6 cells; t7, time to cleavage into 7 cells; t8, time to cleavage into 8 cells; cc2, duration to the second cleavage; s2, synchrony of the second cell cycle; cc3, duration to the third cleavage; s3, synchrony of the third cell cycle. The Wilcoxon sum ranked test was used for comparing between the study and control groups. Data is presented as median and interquartile range (Q1, Q3)*.

** *All morphokinetic parameters are expressed in relation to tPNF (time of pronuclear fading)*.

**Figure 2:**
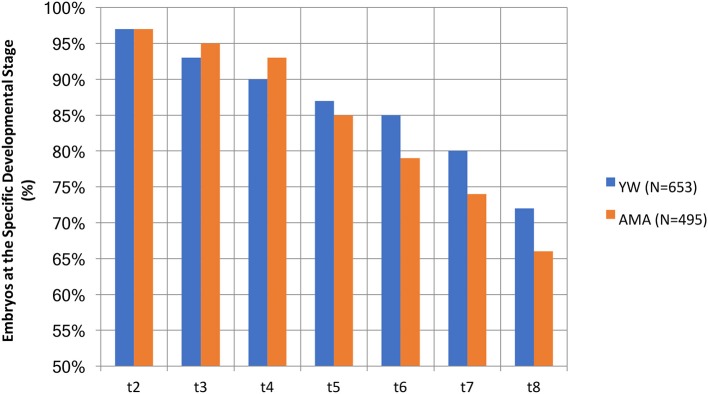
A comparison of the percentage of AMA (study) and YW (control) embryos existing at the specific developmental stage.

We also analyzed the developmental stage to which embryos of the two groups progressed. It is worth noting that the AMA embryos appeared more prone to developmental arrest compared to the YW group's embryos (Figure 2); specifically, only 66% of the AMA embryos, reached the 8-cell stage, vs. 72% in the younger group (Figure 2), however, these differences didn't reach a statistical significance.

## Discussion

The relationship between female age and embryo morphology using static evaluations has been explored in a number of studies which demonstrate that the routinely used morphological scoring systems, such as blastomere count, cleavage patterns, and degree of fragmentation reflect no age-related effect on oocyte and embryo quality (19–21). The current study used morphokinetic analysis to assess whether this higher-resolution embryo scoring system could identify kinetic parameters and/or dynamics unique to the cohort of older women.

With over 1,000 embryos, this study is the largest and first systematic analysis of the morphokinetic parameters of embryos from women ≥42 years of age and their comparison with embryos of younger women. In addition, this study focuses on the female factor while neutralizing the impact of male factor, as it includes only embryos created via standard IVF, while excluding those produced via ICSI. Although, the embryos of older women appeared to be more prone to developmental arrest prior to the 8-cell stage, these differences did not prove significant. In addition, no significant differences were observed in the morphokinetic parameters during the first 3 days of pre-implantation development between embryos of women of AMA and those of younger women Our results are in accordance with Gryshchenko et al. (30) whose study analyzed various factors influencing embryo morphokinetics and demonstrated that age had no significant effect on the kinetic parameters of the first 3 days of embryo development. Akarsu et al. (31) demonstrated limited age-related significant delays in a number of kinetic variables (tPNf, t2, t3, and t4) of embryos produced by women aged 30–40 years of age when compared to younger women (20–30 years) (31). Both studies, however, included a limited sample size and were based on a cohort of women below the age of 40, whereas the current study includes a larger sample of women/embryos of older women >42 years.

Interestingly, a recent retrospective study assessed the impact of confounding factors such as maternal age on time-lapse algorithms (32). Their result show that embryos with similar morphokinetic grading of women above 35 years had a significantly lower implantation rate compared to those of women below 35 years. These findings question the role of morphokinetics alone in predicting embryo outcome, suggesting that the fate of older embryos is more likely a result of their genetic/chromosomal constitution. This study, however, compared women below and above the age of 35, while our study is unique in analyzing a group of AMA women above 42.

Our results presented here demonstrate that embryos of older women appear to be more prone to developmental arrest through the five to eight cell stages (only 66% of AMA embryos reached the 8 cell stage as compared to 72% of the younger women). This finding can explain their negative prognostic factor for implantation, since an analysis of implanted embryos (KID-positive embryos) proposed that embryos with fewer than 8 cells at 68 h post-insemination might serve as a qualitative parameter for their de-selection for transfer (26). Although, the increased developmental arrest among AMA embryos in this study did not reach a statistical significance when compared with YW embryos, we assume that if a similar morphokinetic study was to be performed in which embryos would be cultured until day 5, it is likely that significant delays in later morphokinetic parameters, such as start of blastulation, would be observed in embryos of AMA women. This assumption is based on the results of this study and the results of others showing a decreased rate of blastocyst formation and decreased pregnancy rates among AMA women (33, 34). Indeed, the robust literature demonstrates a clear relationship between increased age and decreased pregnancy and delivery rates (3–7) and the very low live birth rate of AMA patients in our study is in line with existing data. The median morphokinetic parameters of embryos observed in both the AMA and YW groups in this study, including t5, s2, cc2, and cc3 correlate with the normal ranges found to be associated with implantation. Using a logistic regression model, cc2, s2, and t5 were defined as important predictors for implantation success (24, 35). Another study developed an algorithm for embryo selection and identified cc3 as the morphokinetic parameter most significantly associated with implantation (22).

It is also well-accepted that the incidence of chromosomal aneuploidy increases with maternal age, with up to 70–80% of embryos from women 38–42 years of age demonstrating chromosomal abnormalities (10). Numerous studies suggest that early morphokinetic parameters may offer hints regarding chromosomal ploidy; some suggest that early morphokinetic blastomere behavior may be indicative of ploidy, while an additional study observed differences between aneuploid and euploid embryos only in the peri-blastulation stage (36–38). Alternatively, two large studies by Yang et al. and Rienzi et al. which combined TLM and comparative genomic hybridizations analysis failed to detect any correlation between blastocyst aneuploidy and morphokinetics (39, 40). Thus, while reasonable to expect that early embryonic markers may reflect quality differences between the two groups, it would appear that caution must be exercised in utilizing morphokinetic parameters to select for euploidy, and that further large-scale studies must be conducted to clarify this possible correlation.

Our study presents the following limitations. First, our analysis was limited to morphokinetic markers that comprise the first 3 days of development and did not include the periblastulation and blastulation stages, given that in our unit the policy is to transfer embryos of AMA women on day 3. However, it is reasonable to predict that if embryos of both the AMA and YW women would be grown *in vitro* up to the blastocyst stage, the timing of compaction, the start of blastulation and the level of developmental arrest would be even more greatly affected by age. Second, our study does not assess the incidence of direct cleavage or reverse cleavage, phenomena, which while quite rate, may affect implantation potential. A study comprising an even larger group of embryos would be necessary in order to explore a correlation between abnormal cleavage and female age. Third, given the increasing rate of aneuploidy with AMA, it is worth further exploring the correlation between morphokinetics and ploidy, an area on which preliminary studies present mixed conclusions. In addition, while we reported the overall pregnancy rates of the treatments included in the study, it was impossible to associate the morphokinetic variables of individual embryos with their implantation data, since it is quite rare to transfer single embryos to AMA women. Therefore, in the case of a positive singleton pregnancy, it is impossible to know which of the transferred embryos had been successfully implanted. For this reason, the construction of a KID-positive group of AMA embryos, which would undoubtedly be an invaluable addition to this body of research, was not possible at this time, and reflects a possible direction for future research. A final limitation is the study's retrospective nature, which was unable to control for potential confounding factors (both observed and unobserved) such AMH levels, number of attempts, etc.

Despite these limitations, our study reflects the first large scale study attempted to identify morphokinetic parameters and dynamics unique to AMA women. Such studies can guide the selection of embryos with an increased implantation potential and thus contribute to enhanced outcomes among these women.

## Data Availability Statement

The datasets generated for this study are available on request to the corresponding author.

## Ethics Statement

The studies involving human participants were reviewed and approved by The Ethics Committee of Tel Aviv Medical Center, and institutional review board approval for retrieving IVF data was obtained (0606/17). Written informed consent for participation was not required for this study in accordance with the national legislation and the institutional requirements.

## Author's Note

This work is submitted as part of the requirements for Medical Doctor degree (MW) at the Sackler Faculty of Medicine, Tel-Aviv University.

## Author Contributions

MW and DB-Y: substantial contributions to conception and design, analysis and interpretation of data, drafting the article, and final approval of the version to be published. YK, NS, MD, and FA: acquisition of data, revising it critically for important intellectual content, and final approval of the version to be published. AC: acquisition of data and final approval of the version to be published.

### Conflict of Interest

The authors declare that the research was conducted in the absence of any commercial or financial relationships that could be construed as a potential conflict of interest.
