# Supplementary material for: The Effect of Advanced Maternal Age on Embryo Morphokinetics
Source: Front Endocrinol (Lausanne). 2019 Oct 25;10:686. doi: 10.3389/fendo.2019.00686 (PMC6823873; doi:10.3389/fendo.2019.00686)

**Supplemental Figure 1**
**Participants Inclusion / Exclusion Criteria**

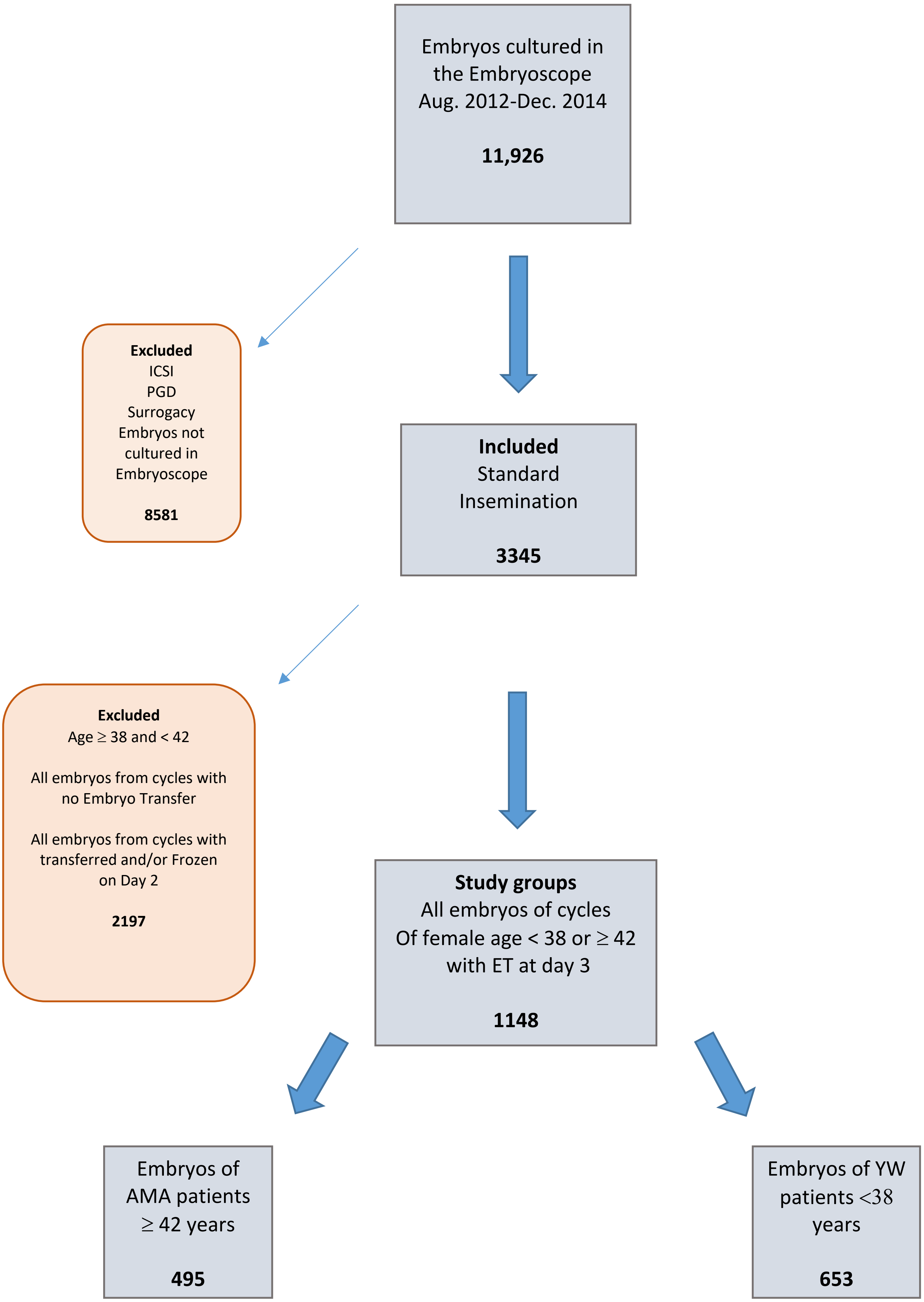

**Supplemental Figure 2**
**Study Age Demographics**

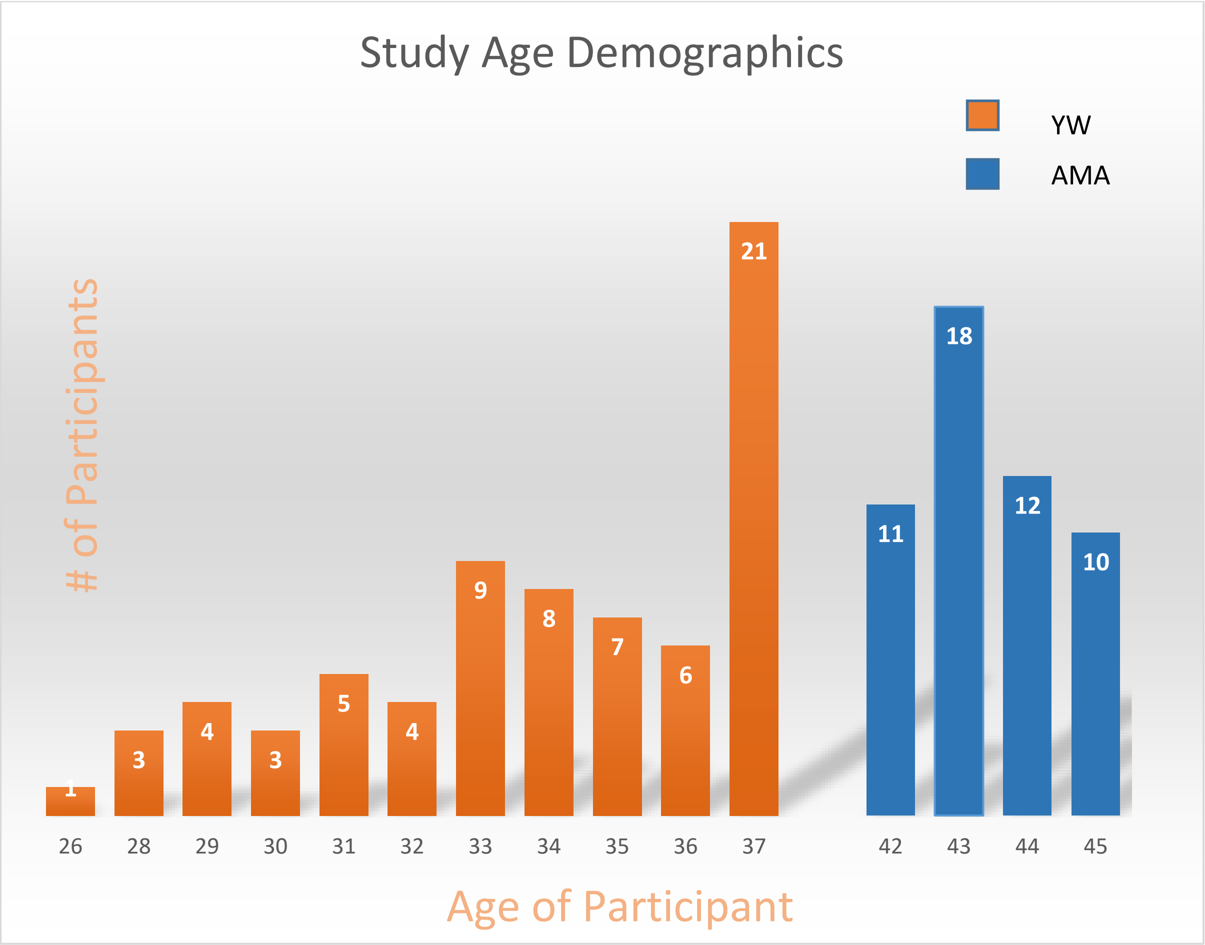

Supplement: Supplementary file 1 [file Data_Sheet_1.pdf]
